# Mobilization in Neurocritical Care: Challenges and Opportunities

**DOI:** 10.1007/s11910-024-01399-y

**Published:** 2024-12-26

**Authors:** Kristen Nobles, Kyle Cunningham, Brianna Fecondo, Susan M. Closs, Kathleen Donovan, Monisha A. Kumar

**Affiliations:** 1 Department of Neurology, Hospital of The University of Pennsylvania and Penn Presbyterian Medical Center, Perelman School of Medicine, University of Pennsylvania, Philadelphia, PA USA; 2 Department of Physical and Occupational Therapy, Hospital of The University of Pennsylvania and Penn Presbyterian Medical Center, Perelman School of Medicine, University of Pennsylvania, Philadelphia, PA USA; 3 Department of Neurosurgery, Hospital of The University of Pennsylvania and Penn Presbyterian Medical Center, Perelman School of Medicine, University of Pennsylvania, Philadelphia, PA USA; 4 Anesthesiology & Critical Care, Hospital of The University of Pennsylvania, Philadelphia, PA USA

**Keywords:** ICU mobilization, Neurologic intensive care unit, NICU mobilization, Neurocritical care, Neurologic injury, Falls risk, Delirium

## Abstract

**Purpose of Review:**

Mobilization in the Neurological Intensive Care Unit (NICU) significantly improves outcomes and functional recovery while preventing immobility-related complications. The heterogeneity of neurologic conditions necessitates tailored, interdisciplinary mobilization strategies. This article reviews recent research on enhancing the feasibility and effectiveness of mobilization interventions in NICU settings.

**Recent Findings:**

Early mobilization improves functional outcomes, reduces complications like muscle atrophy and pressure ulcers, and can shorten ICU stays. Safe implementation involves individualized protocols and a multidisciplinary team, emphasizing that early mobilization benefits critically ill neurological patients.

**Summary:**

Development of evidenced-based protocols for interdisciplinary NICU patient mobilization enhances patient outcomes and quality of life. Use of outcome measures can facilitate mobility while preventing complications from immobility. Future research in embracing emerging technologies such as mobilization equipment and virtual/augmented reality will help determine optimal timing as well as dosage of mobility to improve long-term functional outcomes in the unique NICU population.

## Introduction

The Neurological Intensive Care Unit (NICU) serves patients with critical neurological illnesses. Patients often experience profound physical and cognitive impairments, necessitating intensive monitoring and management. Intensive care unit (ICU) mobilization has gained recognition as a crucial component of care in the NICU. The practice aims to prevent complications associated with immobility, promote neuroplasticity, enhance functional recovery, reduce ICU length of stay, and improve clinical outcomes [1, 2].

ICU mobility can be defined as the initiation of movement that progresses a patient towards functional independence. Effective mobilization in the NICU requires the concerted efforts of a multidisciplinary team, including physicians, advanced practice providers, nurses, physical therapists, occupational therapists, and respiratory therapists [3]. Each professional contributes specialized knowledge and skills to the assessment, planning, and implementation of mobilization strategies. Interprofessional communication and coordination are vital to integrate early mobility seamlessly into the comprehensive care plan, ensuring that individual patient needs and preferences are addressed.

The heterogeneity of neurological conditions and resultant deficits pose unique challenges in the NICU, necessitating a personalized approach to mobilization. Factors such as the severity of the neurological injury, comorbidities, cognitive function, verbal comprehension, pre-morbid functional status, and psychosocial support influence the practicality and appropriateness of mobilization interventions. Mobilization has significant potential to optimize outcomes for patients with neurologic injury in the intensive care setting. Innovative approaches, interdisciplinary collaboration, and tailored care strategies present opportunities to overcome challenges while enhancing the feasibility and effectiveness of mobilization interventions in the NICU.

## Mobilization in the Neuro ICU

### Challenges and Opportunities: Medical

Mobilizing patients in the NICU presents unique challenges specifically due to the complex pathophysiology of neurological conditions. Medical management of the NICU patient not only involves monitoring physiologic parameters but requires close attention to cognitive status.

#### Physiologic Barriers

When considering ICU mobilization, patients with neurologic injuries pose distinctive challenges due to their dynamic and complicated disease process. Patients often exhibit altered consciousness, impaired cognition, compromised comprehension, motor dysfunction, hemodynamic instability, and increased susceptibility to secondary neurological injuries, necessitating a cautious approach to minimize the risk of adverse events [3–5]. Determining eligibility for mobilization in these patients requires careful consideration of patient-specific factors [6]. Establishing standards for mobilization regarding timing, dose, frequency, and duration remains challenging due to heterogeneous trials and the lack of well-established protocols.

When considering medical factors that influence the dosage or timing for mobility, one must account for the patient’s primary neurological injury, potential for secondary injury, and other medical conditions resulting from their neurological injury. Mobility that is too intense or initiated too early after a neurological injury may provide no benefit and could be detrimental [6–8]. Resolution of shock and intracranial pressure (ICP) crises are imperative prior to ICU mobility. Additionally, ensuring adequate perfusion to the central nervous system helps mitigate the risk of ischemic events [9]. Traditional management of intracranial hypertension such as: hyperosmolar therapy, cerebral spinal fluid diversion, hyperventilation, hypothermia, analgesia, sedation, barbiturate coma, and decompressive surgery, come with significant side effects and limitations to mobilization [10–19].

Patients with spinal cord injury (SCI) serve as a prime example of such complexity. These patients may have unstable injuries requiring early surgical stabilization. The sequelae of SCI, such as respiratory failure, bradycardia, hypotension, and neurogenic shock, must be well managed before mobilization can be safely initiated. In some cases, this necessitates interventions like temporary pacemaker placement [20] and tracheostomy insertion [21–23]. Proper management of these conditions is crucial to mitigate the risks associated with mobilization.

The heterogeneity of trials and the lack of well-established protocols make it difficult to standardize mobilization practices. Each patient’s unique medical condition necessitates a tailored approach to determine the appropriate timing, dose, frequency, and duration of mobilization activities. For instance, the mobilization of patients with traumatic brain injury (TBI) must consider the risk of increased intracranial pressure with careful assessment of neurologic status to avoid exacerbating injury [24–26]. Protocols for patients with SCI may involve staged mobilization strategies starting with passive range of motion exercises, progressing to active assisted movements once hemodynamically stable [7]. This progression helps to prevent complications such as deep vein thrombosis (DVT) and muscle atrophy while ensuring patient safety.

Although there are medical challenges for mobilization in the NICU, current evidence demonstrates this practice is safe and beneficial. Verticalization and mobility have shown promise in improving functional outcomes in neurologic populations [27, 28]. In addition to functional benefits, mobility can aid with many goal-directed therapies. Use of specialty beds not only facilitates mobility, but also treats medical issues like intracranial hypertension. Studies have shown that patients with refractory intracranial hypertension who were treated with verticalization had lower ICP, fewer ICP spikes, and decreased total number of medical interventions needed after verticalization compared to prior [29]. With proper preventative measures such as compression stockings and abdominal binders to mitigate orthostatic hypotension, verticalization has been well tolerated in this patient population, with cautious attention to pressure wounds [29, 30]. Integrating verticalization early in the treatment of intracranial hypertension may help mitigate the side effects seen with traditional treatments.

Mobilizing patients in the NICU is fraught with medical and physiological challenges that require a nuanced and individualized approach. Understanding the complex interplay between neurological injuries and systemic medical conditions is crucial for developing safe and effective mobilization protocols. Ongoing research and clinical trials are essential to establish evidence-based guidelines that can standardize practices and improve patient outcomes in the NICU. By carefully considering patient-specific factors and potential risks, healthcare providers can optimize mobilization strategies to enhance recovery while minimizing the likelihood of adverse events.

#### Cognitive Challenges

Cognition can be defined as the process of acquiring or maintaining knowledge through the interpretation of thoughts, prior experiences, and various sensory inputs. It encompasses multiple domains, including memory, attention, arousal, affect, language, and executive function, to name only a few. Neurologic disorders are the leading cause of cognitive disability [31]. Critically ill patients with respiratory failure or shock are at risk of developing long-term cognitive deficits [32].

Patients that present with cognitive impairments or disorders of consciousness in the NICU are especially challenging to mobilize [1]. Patients that are confused, difficult to redirect, or unable to follow commands are more likely to unintentionally remove invasive lines, drains, and monitors; therefore, constant vigilance is necessary when attempting mobility in this setting. Some may even require the use of physical restraints to prevent the disruption of necessary medical equipment, further inhibiting a patient’s likelihood to mobilize out of bed thus leading to deconditioning [33]. When considering all factors, many providers may be hesitant to mobilize patients out of bed.

Despite these challenges, ICU mobility has been shown to reduce cognitive dysfunction, particularly in critically ill patients that require invasive monitoring or mechanical ventilation [34]. Prompt consultation with physical and occupational therapy can facilitate early mobility in patients that are cognitively impaired. Additionally, therapists can provide cognitive interventions and further recommendations to healthcare staff regarding safe mobilization of these patients, including mechanical lifts or alarm systems. Evidence to support the use of restraints is mixed, with some studies suggesting the risks outweigh the benefits of use [35]. Patients can often still be mobilized out of bed with restraints if a provider can provide direct supervision for a short time, assuming the patient is not combative. In addition, one-to-one supervision can also facilitate early mobility and improve time out of bed. Recent advances in medical technology allow for remote supervision via video monitoring surveillance when in-person staffing is limited, assuming these patients can be redirected easily.

Additionally, patients with unresponsive wakefulness syndrome (formerly known as vegetative state) and minimal consciousness can be mobilized out of bed despite low levels or arousal or awareness. Studies have shown that the use of tilt tables and verticalization beds can improve arousal and consciousness [27, 36]. Outcome measures such as the Coma Recovery Scale-Revised (CRS-R), Coma Recovery Scale Revised-For Accelerated Standardized Assessment (CRSR-FAST), and Motor Behavior Tool-Revised (MBT-r) are validated measures that can detect subtle signs of consciousness and be used to track improvements in consciousness when using mobility interventions in these populations [37–39].

#### Delirium

Delirium is a condition characterized by an acute, significant decline in attention and cognition [40]. This condition is common in adults over the age of 65 in the acute care setting. Delirium can manifest as hyperactive, hypoactive, or a mixture of both [41]. Patients that experience delirium tend to have longer length of stay, increased healthcare costs, earlier loss of independence, and increased morbidity and mortality [42–45]. This becomes even more prevalent in the neurocritical care setting [46, 47]. Providers often choose to reduce light and stimulation in response, either to promote sleep or reduce agitation. Unfortunately, this only hastens the development of delirium. The symptoms of this condition are often missed in neurologic populations, largely because it can be attributed to the patient’s primary neurologic diagnosis [48]. In turn, this often leads to an increase in the frequency of bedside neurologic examinations to assess for changes in patient presentation, further exacerbating delirium. Increased frequency of neurological exams can hasten the development delirium in some neurologic populations [49].

Preventive measures play a crucial role in managing delirium and promoting appropriate sleep cycles. Mobilization out of bed, exposure to light during waking hours, and establishment of a daily routine are fundamental strategies in this regard [50–52]. Collaboration with the multidisciplinary team is essential, particularly in the selection of sedation and analgesics. Continuous sedation should be minimized. Employing the lowest effective dose of analgo-sedation to achieve treatment goals is imperative not only for serial neurological examinations and maintaining alertness but also for preventing delirium [50]. Moreover, the implementation of a delirium bundle focused on promoting sleep-wake cycles for all NICU patients can serve as both a treatment and prevention measure for delirium, facilitating mobilization efforts in the process [50, 52].

Some delirium risk assessment tools can also be beneficial in predicting patients who are at risk for developing delirium. For instance, the AWOL is a mnemonic phrase in which a patient is assigned one point in four different domains; a score of two or more indicates a patient is at high risk for developing delirium [53]. Another measure is the CAM-ICU, which can help identify patients that have already developed delirium so that appropriate interventions may be implemented [54] (Table 1).

Once a patient has been deemed at risk or has already developed delirium, bedside nurses can implement strategies to optimize patient recovery in the NICU. One of the most effective strategies to improve delirium in the NICU is the ABCDEF bundle. When all components of the bundle are used together, it can be an extremely effective tool to prevent delirium. This mnemonic includes the following components [55].

A) Assess, prevent, and manage pain.
B) Both spontaneous awake trials and spontaneous breathing trials should be performed regularly.
C) Choices of analgesics and sedatives are key and should be used sparingly when possible.
D) Delirium should be frequently assessed and managed.
E) Early mobility and exercise facilitate improvements.
F) Family engagement and empowerment are key.

#### Fall Risk

Falls are generally defined as an unintentional event in which a person comes to rest at the ground or a lower-level surface in an uncontrolled manner, excluding certain intrinsic (stroke, syncope) and extrinsic (natural disasters, physical violence) circumstances [56]. Each year, up to one million adults fall in the hospital setting [57]. Falls in the inpatient setting often occur when patients ambulate without required assistance, attempt to perform elimination-related activities, or are mobilized at night [58, 59]. Although falls occur less frequently in the critical care setting, patient harm is reported up to 51% of the time [60]. Patients in the NICU are at particular risk when considering the increased frequency of cognitive and motor deficits in these populations [61–63]. Additionally, the need for invasive monitors, lines, drains, and medical devices in this population presents additional risks if a fall were to occur, including infection and dislodgement.

Initial risk assessments regarding falls are typically deferred to bedside nurses. However, a multidisciplinary approach to fall prevention education may reduce the frequency of falls on neurologic care units [64]. In addition, a multi-faceted approach to mitigate falls risk, including the use of fall-risk bracelets, lowered beds, use of alarm systems, restraints (as needed), and polypharmacy assessment, can be effective at reducing the number of falls on an inpatient neurology unit, whereas many of these interventions are less effective as a stand-alone method [65]. Physical and occupational therapists with advanced skills in the NICU setting are especially useful for preventing falls. Tests such as the Five Time Sit-To-Stand Test (FTSST), Timed Up & Go (TUG), and the Two Minute Walk Test (2MWT) are easy, safe, and reliable measures for assessing falls risk in ambulatory patients in the neuro ICU [66]. Additionally, the Tyndall Bailey Falls Risk Assessment Tool (TB FRAT) is a reliable assessment that specifically measures falls risk in the ICU setting [67].

**Table 1:** Useful tools and measures to facilitate mobility in the NICU

Outcome measure (Acronym)	Domain	Description	Advantages	Pitfalls	Reference
AWOL	Delirium	Mnemonic used to assess patients at-risk for delirium. One point assigned for Age > 80 years, inability to spell WORLD backwards, not oriented to person or place, or moderate to severe illness on nursing screen. Scores of two or greater indicate patient at-risk for delirium.	• Quick screening tool • Easy to perform • Used as a preventative measure for delirium	• Can only be completed by nursing staff for illness severity scores	[54]
Confusion Assessment Method for the ICU (CAM-ICU)	Delirium	Tool used for diagnosis of delirium. It is a shortened version of the longer Confusion Assessment Method (CAM) and does not requires the use of verbal communication.	• Can be completed by multiple disciplines • Easy to perform • Relatively quick to use	• Not used to identify patients at-risk, only identifies patients that have already developed delirium	[55]
Coma Recovery Scale-Revised (CRS-R)	Consciousness	Comprehensive tool that assesses consciousness. Patients are scored in six domains (Visual, Auditory, Motor, Oromotor, Communication, and Attention). Within each domain, a patient can be categorized as unresponsive, minimally conscious, or emerged from minimal consciousness.	• Comprehensive assessment of consciousness • Standardized assessment • Can help to identify patients with low levels of consciousness they may benefit from treatment interventions, such as the tilt table, to improve arousal	• Takes longer time to complete (generally 25–30 min) • Some subscales may be difficult to assess for patients that are orally intubated • Not directly influenced by mobility practice	[38]
Coma Recovery Scale-Revised for Accelerated Standardized Assessment (CRSR-FAST)	Consciousness	Abbreviated version of the CRS-R that includes five core items used to differentiate patients that are not conscious from those that demonstrate minimal consciousness.	• Can be completed at bedside in five minutes • Specifically developed for ICU • Allows therapists to perform quick consciousness assessment without having to sacrifice treatment time • Allows for easier serial assessment • Can help to identify patients with low levels of consciousness they may benefit from treatment interventions, such as the tilt table, to improve arousal	• Not as thorough as the original CRS-R	[39]
Motor Behavior Tool– Revised (MBT-r)	Consciousness	Supplementary clinical tool that assesses subtle motor behaviors and is used to identify residual cognition in patients with DOC. It includes seven items that are positive predictors of cognitive function, and two items that are negative predictors.	• Helps identify subtle signs of consciousness that may not be captured on CRS-R or CRSR-FAST • Positive findings can facilitate more frequent CRS-R or CRSR-FAST assessments • Can help to identify patients with low levels of consciousness they may benefit from treatment interventions, such as the tilt table, to improve arousal	• Should be used as a supplementary tool • Does not differentiate between unresponsive wakefulness, minimal consciousness, or emergence from minimal consciousness	[40]
Functional Status Score for the ICU (FSS-ICU)	ICU Mobility	ICU-specific assessment of mobility that identifies deficits in five domains: rolling, supine-to-sit transfers, unsupported sitting at edge of bed, sit-to-stand transfers, and ambulation. Each item is scored 0–7, with a total possible score of 35. Higher scores indicate improved function.	• Quicker than the Perme ICU mobility scale • Excellent reliability and validity • MCID value for patients in ICU with stroke = 4.2 points	• Can only be completed by PT/OT • Used to measure change in mobility status over course of ICU admission, does not predict risk for falls • Assess mobility only • Cannot use a lift device to complete	[69, 70]
Perme ICU Mobility	ICU Mobility	ICU-specific assessment of mobility status. It includes 15 items. Items 1–9 are assessed via yes/no, items 10–14 are scored based on assistance needed for mobility, and item 15 is scored based on distance walked in two minutes.	• Identifies barriers to mobility in the ICU • Excellent inter-rater reliability • Includes cognitive component	• Can only be completed by PT/OT • Takes longer time to complete (15–60 min) • Not specifically studied in neurologic ICU population • Used to measure change in mobility status over course of ICU admission, does not predict risk for falls	[71]
Tyndall-Bailey Falls Risk Assessment (TBFRAT)	Falls Risk	Reliable and valid measure to predict risk of falls specifically for patients admitted to the ICU.	• Falls risk assessment specifically developed for use in the ICU • Strong interrater reliability and internal consistency • Shown to reduce incidence of falls in the ICU • Can be completed by multiple members of the interdisciplinary team	• It was studied in a single-occupancy ICU room with 1:1 nursing for intubated patients and 1:2 nursing for lower acuity patients, so it may not translate well to other ICUs that are staffed differently	[68]

### Challenges and Opportunities: Surgical

Mobilization in the Neuro Intensive Care Unit (NICU) is essential for enhancing patient recovery and minimizing complications related to prolonged immobility. However, specific surgical challenges exist that can hinder mobilization efforts.

#### Activity Restrictions

Patients with neurologic injury are particularly prone to impulsivity and agitation, increasing the risk of falls and other complications [71, 72]. This risk is amplified in patients who have undergone neurological surgery, such as craniotomy, decompressive hemicraniectomy (DHC), or the placement of an intracranial device. Post-procedural activity restrictions, including bed rest, head of bed level restrictions, and movement precautions due to arterial access can delay mobilization efforts. For example, patients undergoing cerebral angiogram face a risk of arterial bleeding from the access site, especially when timing of mobilization is considered for those with arterial groin access [73].

Neuro-interventional proceduralists have demonstrated the safety and efficacy of using transradial access for diagnostic and interventional procedures compared to the transfemoral site with equal rates of aneurysm securement for both locations [74] The major benefit of transradial access in neurocritical care patients is the reduced need for post-procedure immobility and bed rest restrictions. This advancement allows for earlier and safer mobilization of patients [75].

**Fig. 1:**
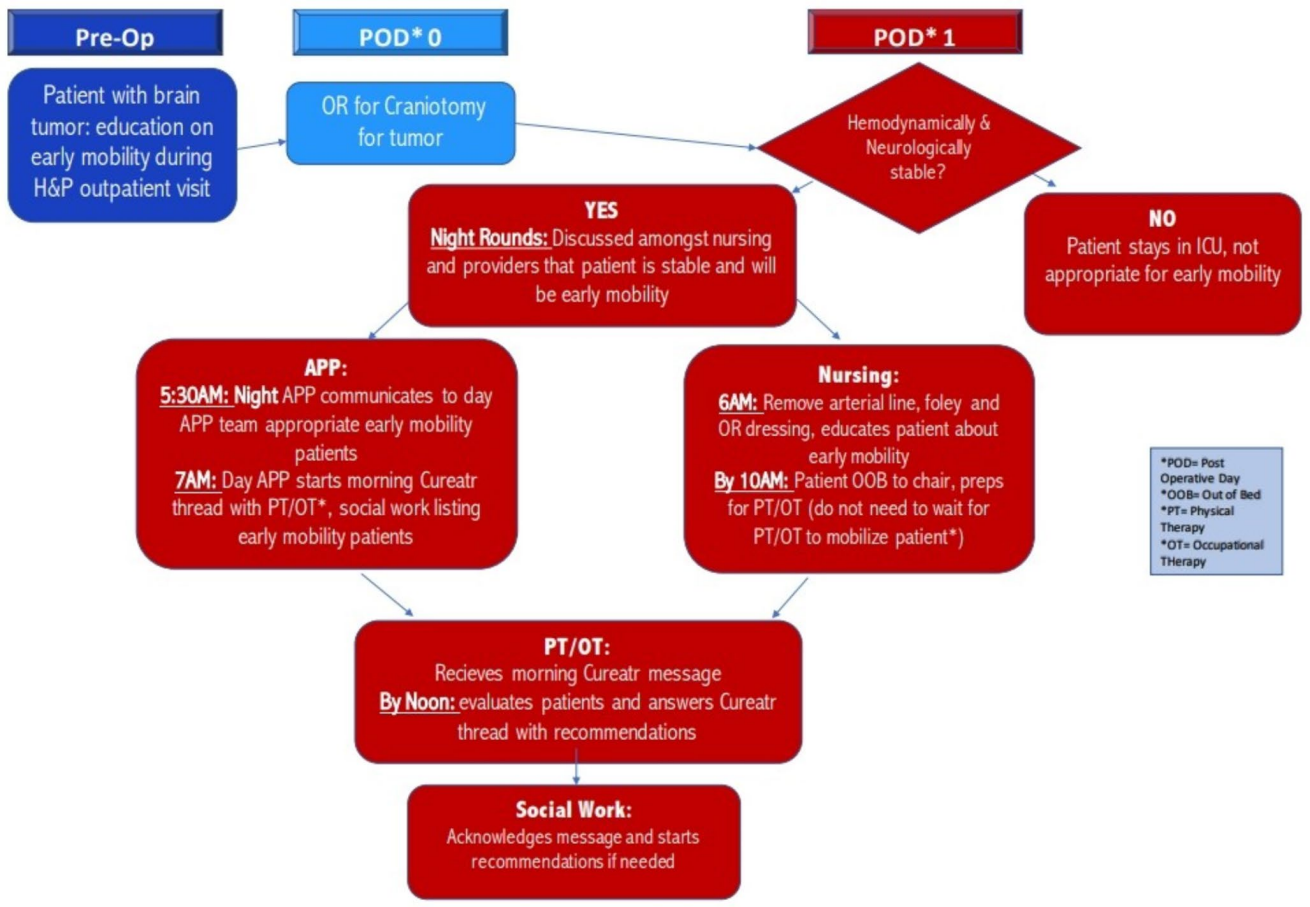
Example mobility protocol for patients status post craniotomy at penn medicine. The redesign and implementation of a protocol to improve early mobility and decrease length of stay in intensive care unit (ICU) patients following elective craniotomy [2021]. Source Permission: Kathryn Kessler, DNP, CRNP

The creation of postoperative mobilization protocols presents the greatest opportunity to improve mobilization in post-surgical patients within the NICU. These protocols enable staff to mobilize patients confidently, while incorporating built-in safety checks. Standardizing utilization of Enhance Recovery After Surgery (ERAS) protocols among the NICU population has been shown to decrease ICU length of stay (LOS), opioid needs, and insulin requirements [76]. ERAS protocols applied specifically to the craniotomy population can expedite postoperative care without increasing complications [77]. Standardized mobilization protocols for patients with craniotomy on the first day post-operatively ensure safe early mobilization while also decreasing ICU LOS (Fig. 1).

#### Invasive Lines and Drains

Invasive devices such as external ventricular drains (EVDs) and lumbar drains pose risks of dislodgement during mobilization, potentially leading to over or under-drainage of cerebrospinal fluid (CSF), altered intracranial pressure (ICP), infection risks, and neurologic decompensation [78–80]. The procedural risk of re-inserting invasive drains after dislodgement must be carefully considered. Additionally, airway devices and feeding tubes, which are common in neurologically injured patients, are at risk of dislodgement during mobilization.

Despite the risks associated with EVDs, research has shown that ICU mobility in this population is beneficial, safe, and productive when compared to standard interventions [81]. Our institution utilizes a specialized EVD mobility protocol that has successfully facilitated safe mobility, utilizing a step-by-step pathway that allows nurses, providers, and therapists to work in unison. Mobility protocols help to prevent negative outcomes associated with EVD placement including venous thromboembolic events, increased time on ventilator and increase ICU and hospital LOS [82]. Additionally, utilization a neurocritical trauma mobility protocols to facilitate mobility in patients with TBI and SCI can help ensure safe and effective mobilization practices, mitigating the risks associated with invasive devices and preventing unwanted complications of prolong immobility.

Addressing surgical challenges and leveraging opportunities can significantly enhance the culture of mobilization in the Neuro Intensive Care Unit. By liberalizing activity restrictions, implementing postoperative mobilization protocols, utilizing transradial access, and safely managing invasive lines, the NICU can improve patient outcomes while hastening recovery. Establishing and adhering to comprehensive mobilization protocols is crucial for fostering a culture of safety and efficiency, ultimately benefiting both patients and healthcare providers.

### Challenges and Opportunities: Cultural

Addressing cultural challenges and leveraging opportunities can transform the culture in the Neuro ICU to support and prioritize patient mobilization. By focusing on stakeholder expectations, leadership commitment, education and training, interdisciplinary collaboration, fear-avoidance behaviors, and continuous monitoring and feedback, the Neuro ICU can achieve better patient outcomes and enhance recovery trajectories.

#### Expectations

The perception of a patient’s readiness for mobilization by the ICU team is a significant challenge. The assessment is often influenced by the patient’s level of arousal or agitation, which in turn affects the staff’s comfort and confidence in initiating mobilization. Variability in comfort levels among staff members can impede mobilization efforts. Enhanced training and experience are essential to build confidence and competence within the ICU team [83]. Patients and their families possess distinct expectations and goals regarding recovery, which can impact their willingness to engage in mobilization efforts. Misalignment between patient/family expectations and clinical objectives can hinder participation. Effective communication about the benefits and realistic outcomes of early mobilization is imperative to reconcile these expectations with therapeutic goals. Addressing the concerns and aspirations of patients and families can foster a cooperative approach to mobilization [84].

#### Leadership Commitment and Education

Leadership commitment plays a pivotal role in shaping the culture towards mobilization. A leadership team that visibly values and demonstrates a commitment to ICU mobilization can significantly influence the unit’s culture. The development and advocacy of policies and protocols that prioritize early mobilization, along with providing necessary resources and support, are critical. Leadership can drive cultural change, establishing mobilization as the standard of care within the Neuro ICU [85]. Ongoing education and training programs for the ICU team are vital to enhance their skills, knowledge, and comfort levels regarding patient mobilization. This can result in a more confident and competent workforce. Similarly, educating patients and their families about the benefits and importance of early mobilization can align their expectations with clinical goals, thereby enhancing their participation and support [86].

#### Fear-Avoidance Behaviors

Fear-avoidance behaviors are characterized by exaggerated negative perceptions of an activity, which limits a person’s willingness to participate in it [87]. This may start a vicious cycle that leads to more avoidance, catastrophizing, and unnecessary hypervigilance, further exacerbating disability. Such behaviors can be experienced by patients, families, caregivers, and staff alike. The anticipated fear of pain, falling, injury, and failure to meet expectations may reduce the likelihood that a patient will participate in early mobility. Additionally, novice staff members may avoid mobilizing patients with invasive medical equipment or complex presentations in the ICU [88, 89]. Patients demonstrating fear-avoidance behaviors are more likely to have extended hospital and ICU stays, which can create distrust between providers and patients [90–92].

To quell these fears, it is crucial to provide staff with adequate resources regarding safe patient mobilization. These resources may include hands-on training for staff less experienced with patient mobility, encouraging earlier mobility evaluations from physical and occupational therapists, promoting the use of alarm systems for patients at risk for falls, and developing a non-punitive ‘debrief’ system for staff members when adverse events occur during patient mobility. Mobility champions on nursing units can help facilitate early mobility and provide mentorship to less experienced staff in the Neuro ICU setting [90–92].

#### Interdisciplinary Collaboration

A collaborative approach among various disciplines within the ICU can lead to more cohesive and effective mobilization efforts [3]. Physical and occupational therapists, nurses, and physicians working together can address multiple aspects of patient mobilization. Establishing shared goals and clear communication channels among the ICU team ensures coordinated and consistent mobilization practices. Interdisciplinary collaboration is essential for the successful implementation of mobilization strategies.

#### Monitoring and Feedback

Regularly tracking and reviewing patient outcomes related to mobilization efforts provides valuable feedback for the ICU team. This feedback can identify areas for improvement and highlight successes, fostering a culture of continuous learning and excellence within the Neuro ICU. Implementing a robust system for outcome tracking and utilizing feedback to make continuous improvements in mobilization practices are essential for sustaining progress [93, 94].

### Challenges and Opportunities: Resource Allocation

A multidisciplinary approach to mobility is essential in the ICU. A thorough and comprehensive history, which can be completed by any member of the interdisciplinary team, can help detect patients at risk for falls. Patients with a history of falls during the prior year, or those with certain diagnoses such as stroke and dementia, are at a particularly high risk for falls [95]. Bedside neurologic exams, strength screenings, cognitive outcome measures, and falls risk assessments help nurses determine which patients are suitable for early mobility [96].

#### Staffing

ICU nurse staffing models and dedicated time significantly impact the ability of bedside providers to perform tasks required for patient mobilization. The presence of RNs, CNAs, respiratory therapists, and specialized PT and OT teams is crucial for the safe mobilization of complex ICU patients [97]. Physical and occupational therapists are more likely to mobilize patients to a higher level of mobility than nurses in the critical care setting [98]. However, up to two-thirds of ICUs lack a dedicated physical or occupational therapy team [99]. Furthermore, only 2% of licensed physical therapists in the US have a board specialty in neurology, as governed by the American Board of Physical Therapy Specialties (ABPTS). While exact numbers are difficult to calculate, it can be presumed that only a small number of neurology-certified specialist PTs work in acute care, and even fewer have ICU training or experience. Regarding occupational therapy, there is currently no board specialty certification for neurology. The American Occupational Therapy Association (AOTA) does offer neurology fellowship program, however there are only ten such programs in the US. The availability of Neuro ICU specialized therapists is further limited on weekends and holidays. Also, weekend staffing often presents a challenge due to fewer on-site services generally prioritized for patients medically appropriate for discharge.

ICU mentorship programs, which pair experienced clinicians with novice ones, can improve care quality and increase the availability of staff capable of facilitating early mobility. Dedicated mobility champions can also improve early mobility interventions for critically ill patients with invasive medical devices [100]. Mobility algorithms can standardize practice and provide a roadmap for safe mobilization [4, 81]. Disease-specific severity scales, such as the NIHSS (National Institutes of Health Stroke Scale) or GCS (Glasgow Coma Scale), assist physical and occupational therapists in prioritizing patients who are not appropriate for ICU mobility [101, 102]. These scales also help therapists allocate resources efficiently, ensuring that patients who do not require two skilled therapists are seen by a single therapist on weekends, thus expanding coverage.

#### Environmental

Patients with invasive medical devices require dedicated resources from the ICU team. Organizing invasive lines, drains, and breathing apparatuses for safe mobilization is challenging which additionally demands time, education, and expertise with ICU equipment [103]. Furthermore, a lack of adaptive equipment can hinder ICU mobilization efforts. The setup of an ICU room can pose challenges to mobilization, especially for the most critically ill patients. Equipment such as ventilators and dialysis machines take up considerable space. Small, cramped, or shared patient rooms may further limit a patient’s ability to mobilize to their fullest potential.

Technological innovations have also enhanced mobilization practices in the NICU [104]. Specialized equipment such as tilt tables, verticalization beds, standing frames, portable in-bed cycling devices, robotic-assisted devices, and body-weight support systems enable safe and controlled movement of critically ill patients [105]. Continuous monitoring devices provide real-time feedback on physiological parameters, allowing for personalized mobilization protocols. Moreover, virtual reality (VR) and gaming technologies offer engaging rehabilitation modalities that promote patient participation and adherence to therapy regimens in a controlled environment [106]. VR can be particularly useful in environments where space is limited.

#### Capacity Management

Managing capacity in the Neuro ICU is a complex task that involves optimizing the allocation of critical care resources to maximize patient outcomes. One innovative approach is the implementation of protocols that bypass the ICU altogether for certain neurosurgical populations, such as those undergoing craniotomies or transsphenoidal surgeries (TSAs).

A novel opportunity to improve resource allocation involves the development of an OR to PACU to Stepdown Unit protocol. One study showed that implementing a postoperative pathway to bypass the ICU in a standard patient population resulted in a reduced length of stay and no change in readmission rates. A significant percentage (86%) of patients were discharged home under this pathway. The additional benefit of bypassing the ICU was the improved utilization of critical care resources, releasing 0.95 ICU days per patient, or 185 ICU days across the cohort [107].

By adopting such protocols, hospitals can alleviate the burden on ICU beds and staff, allowing for more efficient use of resources. This approach not only improves patient throughput but also ensures that ICU resources are reserved for patients with the highest acuity needs. Establishing clear criteria for patient eligibility and developing detailed postoperative pathways are essential steps in implementing this strategy effectively.

## Conclusion

Addressing the challenges of mobility in neurocritical patients requires an interdisciplinary team and a culture that promotes mobility. A comprehensive understanding of patient-specific factors and the implementation of tailored protocols help to facilitate this. Embracing emerging technologies and rehabilitation strategies offers promising avenues for enhancing patient outcomes and quality of life in this complex patient population. The use of outcome measures can help facilitate mobility and identify patients at risk for developing complications from immobility. Future research endeavors should focus on elucidating the optimal timing and dosing of interventions to maximize efficacy as well as improve long-term functional outcomes.

## Data Availability

No datasets were generated or analysed during the current study.
